# Xpert Ultra Can Unambiguously Identify Specific Rifampin Resistance-Conferring Mutations

**DOI:** 10.1128/JCM.00686-18

**Published:** 2018-08-27

**Authors:** Kamela C. S. Ng, Armand van Deun, Conor J. Meehan, Gabriela Torrea, Michèle Driesen, Siemon Gabriëls, Leen Rigouts, Emmanuel André, Bouke C. de Jong

**Affiliations:** a Unit of Mycobacteriology, Department of Biomedical Sciences, Institute of Tropical Medicine, Antwerp, Belgium; b International Union against Tuberculosis and Lung Disease, Paris, France; c Department of Biomedical Sciences, University of Antwerp, Antwerp, Belgium; d Laboratory of Clinical Bacteriology and Mycology, KU Leuven, Leuven, Belgium; Johns Hopkins University School of Medicine

**Keywords:** Xpert MTB/RIF Ultra, rifampin-resistant tuberculosis, *rpoB* mutations, disputed mutations, Ultra probes, melt peak temperature (*T_m_*), melting temperature shift (Δ*T_m_*)

## LETTER

The deluge of data produced by the Xpert MTB/RIF test (Cepheid) can help improve global rifampin-resistant tuberculosis (RR-TB) control strategies through molecular epidemiological surveillance (1, 2). Recently, a new version of the test, Xpert Ultra (hereinafter called Ultra), was released (3). Determining the relationship between RR-conferring *rpoB* mutations, Ultra probes, and melting temperature shifts (Δ*T_m_*), i.e., the difference between mutant and wild-type melting temperatures, allows Ultra results to be utilized for rapid detection of RR-TB strains and related underlying *rpoB* mutations.

To determine the reliability of Ultra results for predicting specific mutations, we tested 13 rifampin-susceptible (RS)-TB strains and 104 RR-TB strains harboring 33 unique RR-conferring mutations from the Belgian Coordinated Collections of Microorganisms in the Institute of Tropical Medicine Antwerp according to a protocol previously described (2) (see the supplemental material). Of note, the Glu250Gly (*n* = 2) and Arg299Cys (*n* = 1) mutations were among the RS-TB strains. We then compared Ultra raw results with available *rpoB* sequences of the strains.

**FIG 1:**
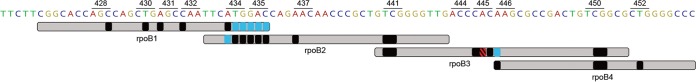
Overview of Xpert Ultra test results. The observed probe reactions for each RRDR mutation were laid over the claimed probe coverage (light gray). Shown in black are probe reactions concordant with manufacturer claims, in blue are probe reactions missed by one probe but captured by another probe, and in red is a probe reaction representing a “RIF Resistance Indeterminate” result from 3 out of 4 strains tested. Results in the hatched pattern were superimposed for greater visibility.

Overall, 29/30 (97%) mutations inside the rifampin resistance-determining region (RRDR) were correctly identified by Ultra. Of concern, mutation His445Arg gave a “RIF Resistance Indeterminate” result among 3/4 strains tested, while it was reported as RR in the initial validation study (3). The silent mutation Thr444Thr was not reported as RR (Fig. 1). The RR-conferring mutations on codons 170 and 491 situated outside the RRDR were not detected.

The probe reactions observed were largely in agreement with previous results (3), although we noted that mutations Met434Val, Met434Thr, and those in codon 435 were captured only by probe rpoB2, Ser450Leu and Ser450Trp were captured by both probe rpoB3 and probe rpoB4a, His445Arg was captured only by probe rpoB3, and Lys446Gln was captured only by probe rpoB4.

**FIG 2:**
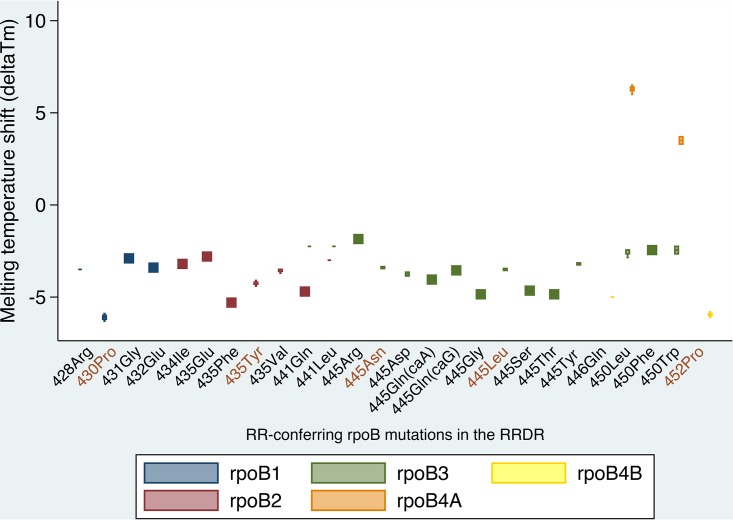
Melting temperature shifts (Δ*T_m_*s) observed upon detection of a rifampin resistance (RR)-conferring *rpoB* mutation in the RR-determining region (RRDR) by Xpert Ultra. The *y* axis reflects the melting temperature difference (Δ*T_m_*) between mutant and wild-type probe-amplicon hybrids, while the *x* axis shows the mutations that we tested. The data points on the graph are Δ*T_m_* values grouped by their associated Ultra probes (differentiated by color), which correspond to a specific *rpoB* mutation. *x* axis labels in brown are disputed mutations.

All mutations except those in codon 450 were associated with a negative Δ*T_m_* (Fig. 2). The combination of Δ*T_m_* values with the capturing probes enabled us to differentiate mutations in codons 430, 431, 434, 435, 441, 446, 450, and 452, including disputed mutations (4) (Table 1). Mutation Asp435Tyr was unambiguously distinguished from Asp435Val with the ∣Δ*T_m_*∣ of probe rpoB2, while mutations Ser441Gln and Ser441Leu were discriminated from the rest by the ∣Δ*T_m_*∣ values of probes rpoB2 and rpoB3. Mutations His445Asp and His445Tyr were distinguished from disputed mutations His445Leu and His445Asn through the ∣Δ*T_m_*∣ of probe rpoB3. Ser450Leu was distinguished from Ser450Trp by the ∣Δ*T_m_*∣ of probe rpoB4A. The indeterminate result associated with His445Arg may be caused by its ∣Δ*T_m_*∣ being equal to 1.8°C, unlike the ∣Δ*T_m_*∣ values for other mutations, which typically exceed 2°C. Our recent experience with Ultra on diagnostic sputum samples pertained only to the Ser450Leu and His445Asp mutations, for which the Δ*T_m_* corresponded exactly with the Δ*T_m_* that we observed for bacterial thermolysates. This should be validated more extensively, which is beyond the scope of our present study.

**TABLE 1:** Xpert Ultra raw results^*a*^

Mutation(s)^*b*^	No. of strains tested	Nucleotide change(s)	Xpert Ultra probe(s)	Wild-type*T*_*m*_ range(s) (mean[s])	Mutant *T*_*m*_ range(s)	∣Δ*T*_*m*_∣ mean(s) or range(s)
Val170Phe	3	GTC→TTC	ND	ND	ND	ND
Glu250Gly^#^	2	GAG→GGG	ND	ND	ND	ND
Arg299Cys^#^	1	CGC→TGC	ND	ND	ND	ND
*Leu430Pro	8	CTG→CCG	rpoB1	69.1–69.5 (69.3)	63.0–63.4	5.9–6.3
*Leu430Pro* + *Met434Ile	1	CTG→CCG; ATG→ATA	rpoB1; rpoB2	69.1–69.5 (69.3); 72.8–73.2 (73)	63.2; 69.8	6.1; 3.2
*Leu430Pro* + Met434Val	1	CTG→CCG; ATG→GTG	rpoB1	69.1–69.5 (69.3)	63.0	6.3
*Leu430Pro* + His445Gln	1	CTG→CCG; CAC→CAG	rpoB1; rpoB3	69.1–69.5 (69.3); 75.5–76.0 (75.75)	63.5; 72.2	5.8; 3.6
*Leu430Pro* + His445Gln	1	CTG→CCG; CAC→CAA	rpoB1; rpoB3	69.1–69.5 (69.3); 75.5–76.0 (75.75)	63.1; 71.7	6.2; 4.1
Asp435Gly + Met434Thr	1	GAC→GGC; ATG→ACG	rpoB2	72.8–73.2 (73)	69.7	3.3
*Asp435Phe	1	GAC→TTC	rpoB2	72.8–73.2 (73)	67.7	5.3
*Asp435Tyr	11	GAC→TAC	rpoB2	72.8–73.2 (73)	68.6–69.0	4.0–4.4
*Asp435Tyr* + Asn437Asp	1	GAC→TAC; AAC→GAC	rpoB2	72.8–73.2 (73)	66.6	6.4
*Asp435Tyr* + Met434Ile	1	GAC→TAC; ATG→ATT	rpoB2	72.8–73.2 (73)	68.5	4.5
*Asp435Val	5	GAC→GTC	rpoB2	72.8–73.2 (73)	69.3–69.5	3.5–3.7
Asp435Val + Gln432Glu	1	GAC→GTC; CAA→GAA	rpoB2; rpoB1	72.8–73.2 (73); 69.1–69.5 (69.3)	70.5; 65.9	2.5; 3.4
*Ser441Gln	1	TCG→CAG	rpoB2; rpoB3	72.8–73.2 (73); 75.5–76.0 (75.75)	68.3; 73.5	4.7; 2.3
*Ser441Leu	1	TCG→TTG	rpoB2; rpoB3	72.8–73.2 (73); 75.5–76.0 (75.75)	70.0; 73.5	3.0; 2.3
His445Gly	1	CAC→GGC	rpoB3	75.5–76.0 (75.75)	70.9	4.9
His445Thr	1	CAC→ACC	rpoB3	75.5–76.0 (75.75)	70.9	4.9
His445Ser	1	CAC→AGC	rpoB3	75.5–76.0 (75.75)	71.1	4.7
His445Ser + *Lys446Gln + Thr444Thr	1	CAC→TCC; AAG→CAG; ACC→ACG	rpoB4B	67.0–67.6 (67.3)	62.3	5.0
His445Asp	3	CAC→GAC	rpoB3	75.5–76.0 (75.75)	71.9–72.1	3.7–3.9
*His445Leu*	2	CAC→CTC	rpoB3	75.5–76.0 (75.75)	72.2–72.3	3.5–3.6
*His445Asn*	2	CAC→AAC	rpoB3	75.5–76.0 (75.75)	72.3–72.4	3.4–3.5
*His445Asn* + *Asp435Glu	1	CAC→AAC; GAC→GAA	rpoB3; rpoB2	75.5–76.0 (75.75); 72.8–73.2 (73)	72.4; 70.2	3.4; 2.8
His445Tyr	4	CAC→TAC	rpoB3	75.5–76.0 (75.75)	72.5–72.6	3.2–3.3
*His445Arg	4	CAC→CGC	rpoB3	75.5–76.0 (75.75)	73.9	1.9
His445Arg + Ser428Arg	1	CAC→CGC; AGC→AGG	rpoB1	69.1–69.5 (69.3)	65.8	3.5
Ser450Phe	1	TCG→TTC	rpoB3	75.5–76.0 (75.75)	71.8	4.0
*Ser450Leu	14	TCG→TTG	rpoB3; rpoB4A	75.5–76.0 (75.75); 67.0–67.6 (67.3)	72.9–73.3; 73.3–73.8	2.5–2.9; 6.0–6.5
Ser450Leu + Thr482Asn	2	TCG→TTG; ACC→AAC	rpoB2; rpoB3; rpoB4A	72.8–73.2 (73); 75.5–76.0 (75.75); 67.0–67.6 (67.3)	69.2–69.5; 73.1–73.3; 73.6–73.7	3.5–3.8; 2.5–2.7; 6.3–6.4
Ser450Leu + Ile491Val	2	TCG→TTG; ATC→GTC	rpoB2; rpoB3; rpoB4A	72.8–73.2 (73); 75.5–76.0 (75.75); 67.0–67.6 (67.3)	70.0; 73.2–73.3; 73.6–73.7	3.0; 2.5–2.6; 6.3–6.4
*Ser450Trp	3	TCG→TGG	rpoB3; rpoB4A	75.5–76.0 (75.75); 67.0–67.6 (67.3)	73.1–73.5; 70.6–71.0	2.3–2.7; 3.3–3.7
Ser450Trp + *Ser431Gly	1	TCG→TGG; AGC→GGC	rpoB3; rpoB4A; rpoB1	75.5–76.0 (75.75); 67.0–67.6 (67.3); 69.1–69.5 (69.3)	73.2; 70.7; 66.4	2.6; 3.4; 2.9
*Leu452Pro	12	CTG→CCG	rpoB4B	67.0–67.6 (67.3)	61.2–61.6	5.7–6.1
*Ile491Phe*	10	ATC→TTC	ND	ND	ND	ND

a Capturing probes, wild-type melt peak temperature (*T*_*m*_) ranges and means, mutant *T*_*m*_ ranges, and absolute values of melting temperature shift (Δ*T*_*m*_) ranges associated with specific *rpoB* mutations in the strains tested and the corresponding nucleotide changes. ND, strains that harbored corresponding mutations outside the RRDR yielded a “RIF Resistance Not Detected” result. *, rifampin resistance-determining region (RRDR) mutation unambiguously identified by unique combinations of Ultra probes and Δ*T*_*m*_s, including disputed ones (in italics). ^#^, rifampin susceptible according to phenotypic testing.

b For double mutants, the high-confidence RR-conferring mutations are underlined (6, 7).

Our findings confirm the ability of Ultra to unambiguously identify a wide range of RRDR mutations. With the unprecedented rollout of Xpert MTB/RIF and associated connectivity solutions, such as DataToCare (Savics, Belgium) and GXAlert (SystemOne, USA) (2), Ultra results may allow us to rule out transmission between RR-TB patients in a specific setting (Fig. S1), distinguish relapse from reinfection (5) (Fig. S2), and resolve discordance between an RR Ultra result and a low-level RS phenotypic result due to a disputed mutation. For such applications, it is key that Δ*T_m_* values are included in the exported results.

## Supplementary Material

Supplemental file 1
