# Supplementary material for: Xpert Ultra Can Unambiguously Identify Specific Rifampin Resistance-Conferring Mutations
Source: J Clin Microbiol. 2018 Aug 27;56(9):e00686-18. doi: 10.1128/JCM.00686-18 (PMC6113491; doi:10.1128/JCM.00686-18)
Supplement: Supplemental file 1 [file zjm999096071s1.pdf]

Supplemental file 1

Processing of rifampicin-resistant tuberculosis thermolysates for Xpert Ultra testing: methodological details

We first measured the DNA concentration of the thermolysates through a Qubit® 2.0 Fluorometer (Invitrogen, USA) following manufacturer's instructions. Then, we calculated the weight of DNA per *Mycobacterium tuberculosis* bacillus that served as divisor for each DNA concentration; the quotient of which was the equivalent colony forming units (CFU) per ml of each thermolysate. We then performed 1:2 and 10-fold serial dilutions of thermolysates with initial concentration of 10^8-10^9 CFU/ml until 10^6-10^7 CFU/ml were obtained respectively. Finally, we prepared a 1:2 mixture of diluted thermolysate and XpertMTB/RIF Ultra sample reagent and dispensed in the cartridge following manufacturer's instructions (2).

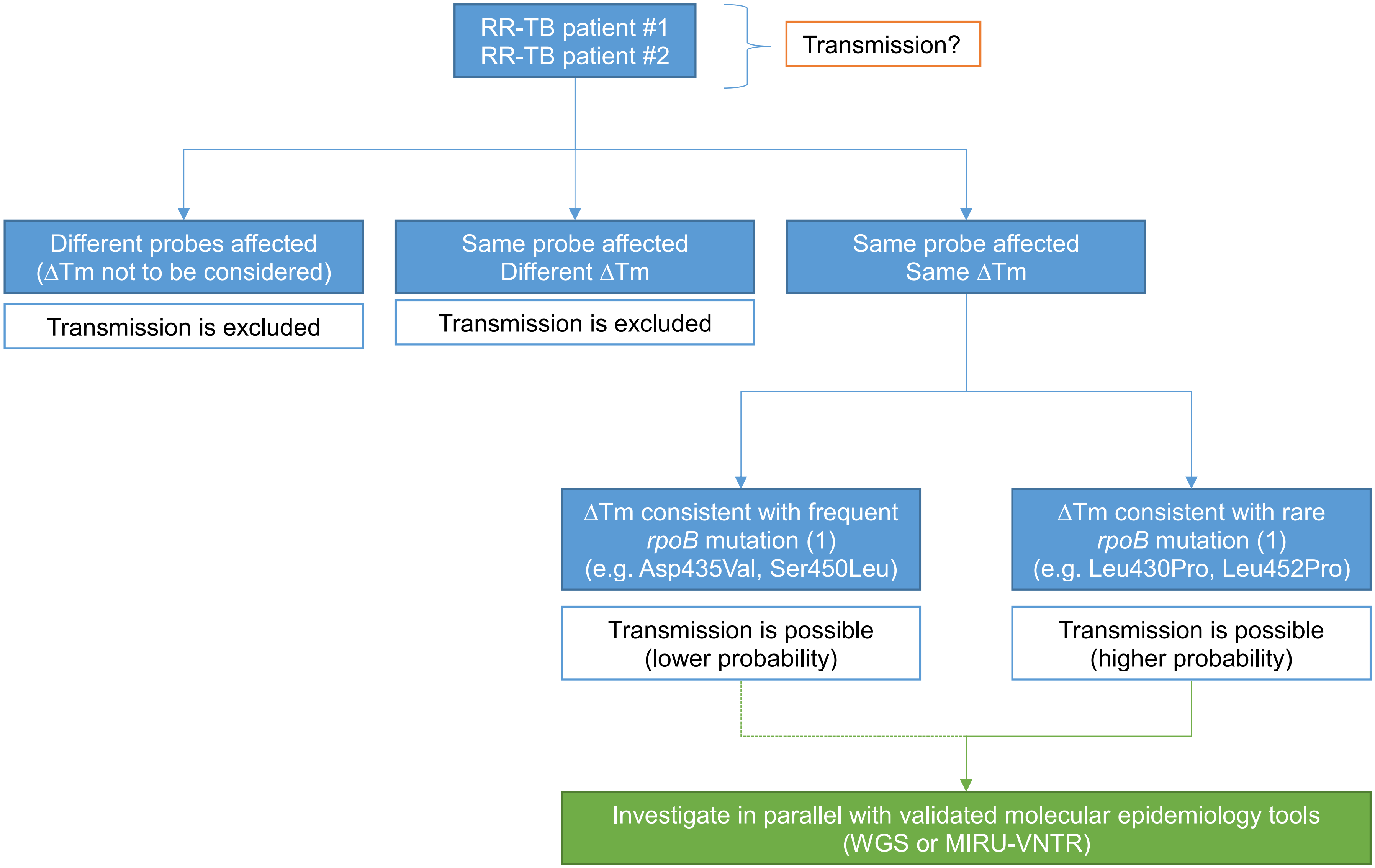

Figure S1. Interpreting Xpert Ultra ΔTm + probe reactions observed in 2 rifampicin-resistant tuberculosis (RR-TB) patients for evaluation of a potential transmission event

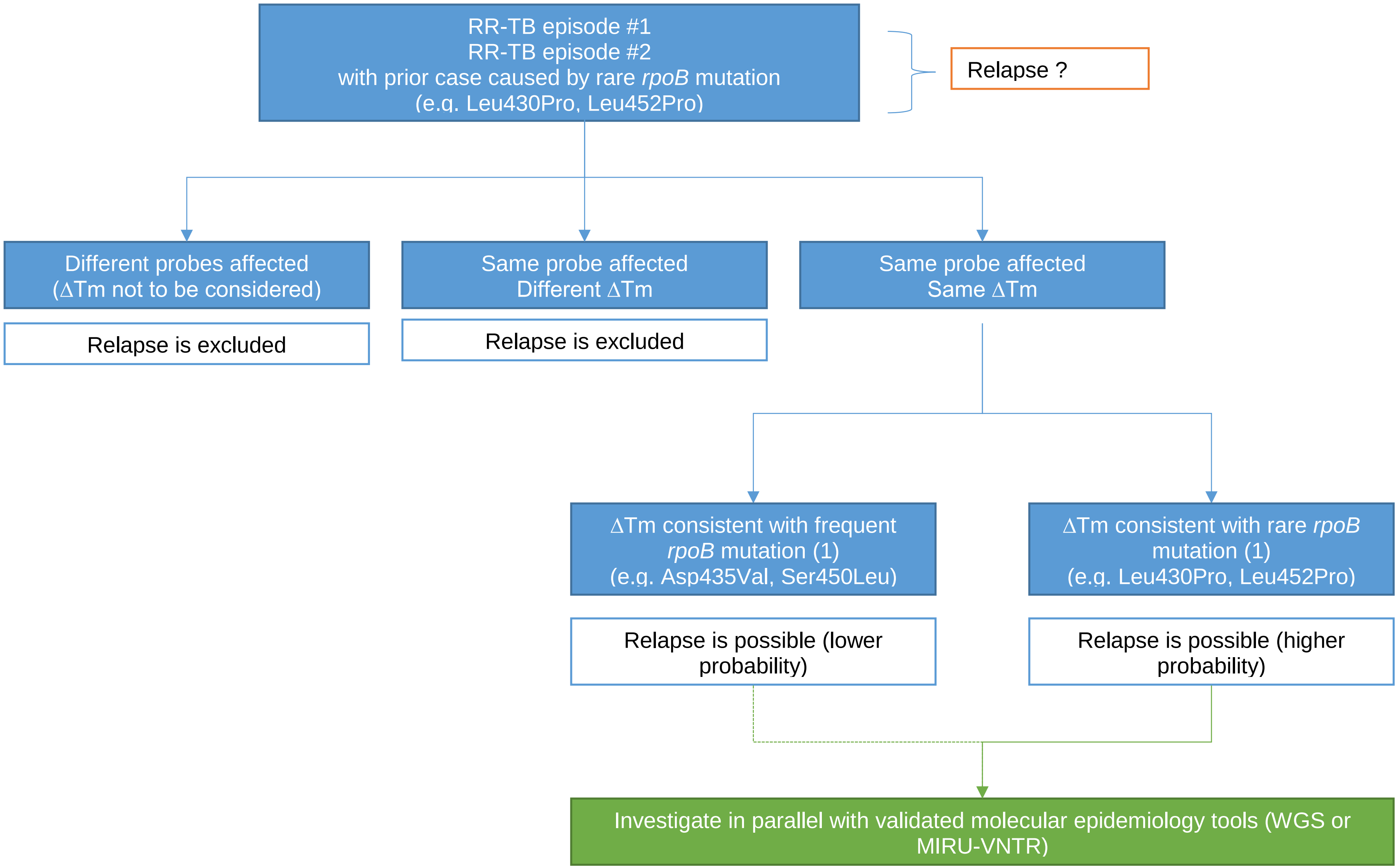

Figure S2. Interpreting Xpert Ultra ΔTm + probe reactions observed in 2 rifampicin-resistant tuberculosis (RR-TB) patients for identification of relapse cases

References

1. Coll F, Phelan J, Hill-Cawthorne GA, Nair MB, Mallard K, Ali S, Abdallah AM, Alghamdi S, Alsomali M, Ahmed AO, Portelli S, Oppong Y, Alves A, Bessa TB, Campino S, Caws M, Chatterjee A, Crampin AC, Dheda K, Furnham N, Glynn JR, Grandjean L, Minh Ha D, Hasan R, Hasan Z, Hibberd ML, Joloba M, Jones-Lopez EC, Matsumoto T, Miranda A, Moore DJ, Mocillo N, Panaiotov S, Parkhill J, Penha C, Perdigao J, Portugal I, Rchiad Z, Robledo J, Sheen P, Shesha NT, Sirgel FA, Sola C, Oliveira Sousa E, Streicher EM, Helden PV, Viveiros M, Warren RM, McNerney R, Pain A, et al. 2018. Genome-wide analysis of multi- and extensively drug-resistant Mycobacterium tuberculosis. Nat Genet 50:307-316.
2. Ng KC, Meehan CJ, Torrea G, Goeminne L, Diels M, Rigouts L, de Jong BC, Andre E. 2018. Potential Application of Digitally Linked Tuberculosis Diagnostics for Real-Time Surveillance of Drug-Resistant Tuberculosis Transmission: Validation and Analysis of Test Results. JMIR Med Inform 6:e12.
